# Ginsenoside Rg1 inhibits dietary-induced obesity and improves obesity-related glucose metabolic disorders

**DOI:** 10.1590/1414-431X20177139

**Published:** 2018-03-01

**Authors:** Jin-bo Li, Rui Zhang, Xiao Han, Chun-li Piao

**Affiliations:** Department of Endocrinology, Affiliated Hospital of Changchun University of Chinese Medicine, Changchun, China

**Keywords:** Diabetes, Ginsenoside Rg1, Insulin resistance, Body weight, Obesity

## Abstract

Obesity and its consequent type 2 diabetes are significant threats to global health. Emerging evidence indicates that ginsenosides from ginseng (*Panax ginseng*) have anti-diabetic activity. We hypothesized that ginsenosides Rg1 could suppress dietary-induced obesity and improve obesity-related glucose metabolic disorders. Our results showed that ginsenoside Rg1 attenuated dietary-induced body weight gain and fat accumulation in white adipocyte tissue of mice fed a high-fat diet. Furthermore, we found that ginsenosides Rg1 not only decreased fasting glucose concentration and the 2-h postprandial glucose concentration, but also improved insulin resistance and glucose intolerance in those mice. Ginsenoside Rg1 also activated the AMPK pathway *in vitro* and *in vivo* and increased plasma membrane translocation of GLUT4 in C2C12 skeletal muscle cells. In conclusion, our observations suggested that ginsenoside Rg1 inhibited dietary-induced obesity and improved obesity-related insulin resistance and glucose intolerance by activation of the AMPK pathway.

## Introduction

Obesity, as a main risk factor for type 2 diabetes (T2D), is caused by accumulation of excess nutrients and excessive expansion of white adipocyte tissue (WAT) (1). Currently, metabolic syndrome and T2D mellitus are increasingly common, primarily because of the prevalence of a sedentary lifestyle and obesity in both developing and developed countries over the last decades (2). Many complications of T2D, such as cardiovascular disease, threaten global health. Obesity-related insulin resistance plays a key role in diabetic pathophysiology (3). Insulin resistance is a lessened capability of insulin to properly activate the insulin signaling pathway that is responsible for the stimulation of glucose uptake and metabolism (4).

Generally, high blood glucose level activates insulin signaling pathway and causes the activity of tyrosine kinase and PI3K/Akt pathway, which further induces translocation of glucose transporter 4 (GLUT4) from an intracellular pool to the plasma membrane (5). Adenosine monophosphate kinase (AMPK), as a master regulator of cellular energy homeostasis, also plays an important role in the regulation of blood glucose level. AMPK also mediates whole-body energy balance by responding to hormones and nutrient signals such as low glucose and hypoxia that lessen the supplies of cellular adenosine triphosphate (ATP) (6). Currently, AMPK is believed to be a therapeutic target of obesity and T2D (7).

Ginseng is considered a tonic and a panacea in traditional Chinese medicine, and has been used to treat many diseases for over 2000 years in Asian countries (8). It is believed that ginseng can improve health and increase metabolism (9). The physiologic and pharmacologic functions of ginseng have been discovered gradually, and include promotion of hematopoiesis, modulation of immune functions, anticancer activity, protection against circulatory shock, and regulation of cellular metabolic processes (9,10). It has been reported that ginseng improves hyperglycemia in animal and human studies (11,12). In addition, ginseng extracts have anti-obesity and anti-hyperglycemic activities in obese animal models (13). Ginseng has been widely studied as alternative medicine for T2D treatment (11). The bioactive compounds of ginseng include polyacetylenes, phenolics, polysaccharides and various ginsenosides (14). Ginsenosides are believed to be the main bioactive fraction of ginseng, and are classified into two groups via the aglycones structures: 20(S)-protopanaxatriol (ginsenosides Re, Rg1, Rg2, Rh1) and 20(S)-protopanaxadiol (ginsenosides Rb1, Rb2, Rb3, Rc, Rd, Rg3) (15). Currently, almost all ginsenosides have been reported to display several bioactivities including anti-ageing, neuroprotective, anticancer, radioprotective, anti-amnestic, and antidiabetic effects (16).

As one of the bioactive ginsenosides, ginsenoside Rg1 has been widely studied. However, the effects of Rg1 on obesity and its related glucose metabolic disorders in animal models are not clear. In the present study, we aimed to evaluate if ginsenoside Rg1 could suppress dietary-induced obesity and improve obesity-related glucose metabolic disorders.

## Material and Methods

### Reagents and antibodies

Ginsenoside Rg1 used in this study was obtained from BTGin Co., Ltd (Okcheon, Korea) and its purity was over 99%. Antibodies for α-IR (insulin receptor), p-α-IR GLUT4, AMPK, p-AMPK(Thr172), and β-actin were purchased from Cell Signaling (USA). Horseradish peroxidase-conjugated anti-rabbit lgG was obtained from sigma (USA). Enhanced chemiluminescence (ECL) reagent was purchased from Millipore (USA). RIPA lysis buffer for protein extraction was purchased from Beyotime Biotech Inc. (China).

### Cell culture and treatment

C2C12 cells were obtained from the American Type Culture Collection (ATCC, CRL-1772). Cells were maintained in Dulbecco modified Eagle's medium (DMEM) supplemented with 10% fetal bovine serum (FBS) and 1% antibiotic (streptomycin and penicillin) in a humidity incubator with 5% CO_2_ at 37°C. DMEM and FBS were obtained from Gibco (USA). For ginsenoside Rg1 treatment, cells were starved by FBS-free and antibiotics-free DMEM overnight. Then, cells were incubated with media containing 1% FBS plus different concentrations of Rg1 (0, 20, and 40 μM).

### Animal studies

Forty-eight 6-week-old C57BL/6J male mice were purchased from Beijing Vital River (China). Mice were fed a high-fat diet (HFD; Research Diets, USA; 60% calories from fat) or a normal diet (ND; 10% calories from fat) as control starting at the age of 7 weeks. HFD mice were randomly divided into 3 groups (n=12 each): controls (isotonic sodium chloride solution, 5 mL/d), mice given 300 mg/kg of ginsenoside Rg1 per day (Rg1-300), and mice given 500 mg/kg of ginsenoside Rg1 per day (Rg1-500). Mice were fed with isotonic sodium chloride solution or ginsenoside Rg1 for 8 weeks using an oral Zonde needle (Natsume, Japan) at 8:00-9:00 am every day. The starting point for treatment was designated as day 0. The animals were all allowed free access to water and diet. Mice body weight was measured weekly. After completion of the treatment, mice were assessed for body weight and WAT weight. All animal studies were approved by the Institutional Animal Care and Use Committee of Affiliated Hospital of Changchun University of Traditional Chinese Medicine.

### Characterization of glucose metabolism

The detections of fasting glucose concentration and postprandial glucose concentration were performed as previously described (9). Fasting glucose concentration was measured after starvation for 12 h at the end of this study. Postprandial glucose concentration was measured at 2 h after refeeding at the end of the study.

The insulin tolerance test (ITT) and oral glucose tolerance test (GTT) were performed after mice treatment with ginsenoside Rg1 for 8 weeks as previously described (17,18). For ITT, mice were injected intraperitoneally with insulin (0.5 U/kg Novolin-R, Novo Nordisk, Denmark). Blood glucose was measured with a one-touch basic glucose meter (Roche, USA) before the injection of insulin and at 20, 40, 60, and 80 min following insulin injection. For GTT, mice were starved overnight, and then orally fed with 1 mg of glucose (20 mg/mL, in water solution) per g of body weight. Blood glucose level was measured with a one-touch basic glucose meter (Roche) before glucose administration and at 20, 40, 60, and 80 min following glucose administration.

### *In vivo* insulin stimulation

Mice were starved overnight at the end of the study. Either saline control or 0.5 U of Novolin-R per kilogram of body weight was administered to mice by tail vein injection. The mice were sacrificed at 5 min after the injection of saline or insulin, and the tissues were isolated and flash frozen in liquid nitrogen.

### Western blot

Membrane protein extraction from C2C12 skeletal muscle cells was carried out by Plasma Membrane Protein Isolation Kit (Abcam, UK) according to the manufacturer’s instructions. Protein extraction and western blot were performed as previously described (19). Briefly, total proteins were extracted from liver and muscle (gastrocnemius muscle) tissues or C2C12 skeletal muscle cells using a RIPA lysis buffer (Beyotime Biotech Inc., China) supplemented with complete EDTA-free protease inhibitor cocktail tablets (Roche) according to the manufacturer’s instructions. The protein concentrations were determined using the BCA Protein Assay Kit (Applygen, China). Proteins (40 μg) were fractionated by gel electrophoresis, transferred to a polyvinylidene fluoride (PVDF) membrane (Millipore), blocked with 5% nonfat milk, and then incubated with primary antibodies overnight at 4°C. After 3 washes with phosphate-buffered saline plus 0.1% Tween-20, the membrane was incubated with horseradish peroxidase-conjugated secondary antibodies for 1 h. Signals were tested by ECL reagent and protein expressions were calculated by normalization to β-actin.

### Statistical analyses

Data are reported as means±SD or SE. The difference between groups was analyzed using Student’s *t*-test when comparing only two groups or one-way analysis of variance (ANOVA) when comparing more than two groups. All statistical calculations were carried out using SPSS 19.0 software (USA) and P<0.05 was considered to be statistically significant.

## Results

### Ginsenoside Rg1 protected mice from dietary-induced obesity

**Figure 1.**
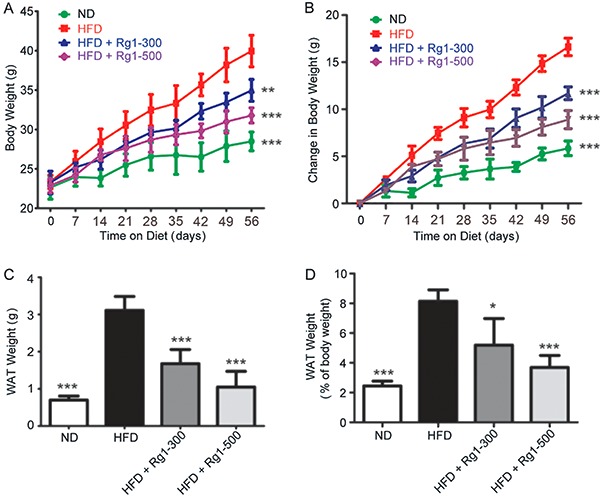
Effect of ginsenoside Rg1 on dietary-induced body weight gain. The high-fat diet (HFD)-fed mice were treated with or without ginsenoside Rg1 at a dose of 300 mg·kg^-1^·day^-1^ (Rg1-300) or 500 mg·kg^-1^·day^-1^ (Rg1-500) for 2 months (n=12 mice/group). *A*, Body weight of mice measured every week, reported as the mean body weight±SE. *B*, Body weight gain every week, reported as means±SE. *C*, Total white adipocyte tissue (WAT) weight and (*D*) total WAT weight/body weight was measured (n=12 mice/group) at the end of the experiment. ND: normal diet. *P<0.05, **P<0.01, ***P<0.001, *vs* HFD group (ANOVA).

Body weight gain was lower in ginsenoside Rg1-treated groups (Rg1-300 and Rg1-500) than in control group (Figure 1A and B and Table 1). The difference of body weight gain was apparent in the 2 weeks and persisted to the end of the study. The body weight gain of HFD-fed mice treated with ginsenoside Rg1 was 29.5% (Rg1-300, P<0.01) and 46.4% (Rg1-500, P<0.01) lower than that of the untreated HFD-fed mice, which suggested that ginsenoside Rg1 treatment prevented dietary-induced body weight gain.

**Table 1.** Body, white adipocyte tissue (WAT), liver, heart, lung, and kidney weight of high-fat diet (HFD)-fed mice.

	ND	HFD	HFD+Rg1-300	HFD+Rg1-500	P-value^a^	P-value^b^	P-value^c^
Initial body weight (g)	23.1±1.0	23.3±0.8	23.3±1.2	23.3±1.2	ns	ns	ns
Final body weight (g)	28.5±1.0	39.9±1.7	34.9±1.2	31.8±0.9	P<0.001	P<0.001	P<0.001
Body weight gain (g)	5.4±0.8	16.6±0.8	11.7±0.7	8.9±0.9	P<0.001	P<0.001	P<0.001
WAT (g)	0.70±0.103	3.11±0.32	1.68±0.33	1.05±0.36	P<0.001	P<0.001	P<0.001
Liver (g)	0.80±0.11	1.20±0.16	0.97±0.12	0.87±0.12	P<0.001	P<0.001	P<0.001
Heart (g)	0.130±0.007	0.132±0.005	0.134±0.006	0.131±0.005	ns	ns	ns
Lung (g)	0.140±0.014	0.143±0.009	0.141±0.007	0.142±0.007	ns	ns	ns
Kidney (g)	0.180±0.012	0.181±0.015	0.183±0.016	0.185±0.011	ns	ns	ns
Fasting glucose (mg/dL)	91.8±11.9	151.4±16.2	119.0±8.0	99.0±6.6	P<0.001	P<0.001	P<0.001

Seven-week-old male C57BL/6J mice were fed with either ND (normal diet) or HFD (high-fat diet). The HFD-fed mice were treated with or without Rg1 at a dose of 300 mg·kg^-1^·day^-1^ (Rg1-300) or 500 mg·kg^-1^·day^-1^ (Rg1-500) for 2 months. Initial and final body weight of mice was measured. The weight of liver, heart, lung, and kidney as well as fasting glucose was measured. Data are reported as means±SE (n=8).

a Comparison between ND group and HFD group;

b Comparison between HFD group and HFD + Rg1-300 group;

c Comparison between HFD group and HFD + Rg1-500 group (one-way ANOVA followed by Dunnett’s post hoc test). ns: not significant.

### Ginsenoside Rg1 protected mice from dietary-induced fat accumulation

As observed in Figure 1C and D and Table 1, the weight of visceral WAT was significantly lower in ginsenoside Rg1-treated mice than that in HFD group (P<0.01). However, the weight of other organs including heart, lung and kidney was unaffected in ginsenoside Rg1-treated HFD-fed mice. Taken together, these results suggested that ginsenoside Rg1 effectively prevented dietary-induced obesity development by inhibiting accumulation of WAT of HFD-fed mice.

### Ginsenoside Rg1 mitigated obesity-induced glucose metabolic disorders

**Figure 2.**
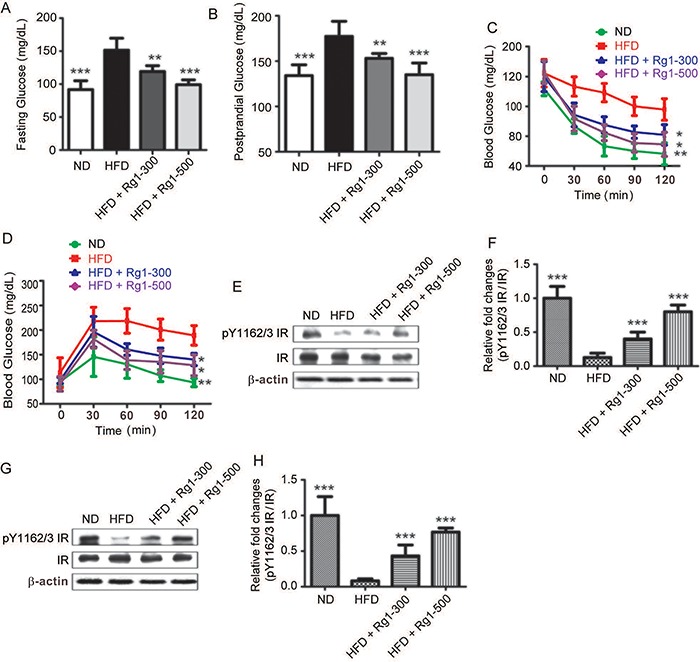
Effect of ginsenoside Rg1 on glucose homeostasis. The high-fat diet (HFD)-fed mice were treated with or without ginsenoside Rg1 at a dose of 300 mg·kg^-1^·day^-1^ (Rg1-300) or 500 mg·kg^-1^·day^-1^ (Rg1-500) for 2 months (n=12 mice/group). The fasting glucose concentration (*A*) and the 2-h postprandial glucose concentration (*B*) as well as insulin tolerance test (ITT) (*C*) and glucose tolerance test (GTT) (*D*) were detected in ginsenoside Rg1-treated and -untreated groups at the end of this study. Immunoblotting assays detected basal protein and phosphorylation levels of inslulin receptor (IR and Py1162/3IR) in liver (*E*) and muscle (*G*). Results are representative of 2 of 12 mice per group. The quantified data are shown in (*F*) for liver tissue detection and (*H*) for muscle tissue detection. ND: normal diet. Data are reported as means±SD. *P<0.05, **P<0.01, ***P<0.001, *vs* HFD group (ANOVA).

Obesity and adipocyte dysfunctions are tightly associated with glucose metabolic disorders (18). Compared with the ND mice, fasting glucose concentration and 2-h postprandial glucose concentration of HFD mice were significantly increased, but these effects were not observed in ginsenoside Rg1-treated HFD mice (Figure 2A and B). Compared with the ND group, HFD-fed mice developed glucose intolerance and insulin resistance. The glucose concentration at 120 min of the ITT and GTT were lower in HFD + Rg1-300 group (P<0.05) and HFD + Rg1-500 group (P<0.01) than in HFD group (Figure 2C and D). Take all together, our observations suggested that ginsenoside Rg1 protected mice from glucose metabolic disorders that caused by dietary-induced obesity.

### Insulin signaling was enhanced in liver and skeletal muscles in ginsenoside Rg1-treated HFD mice

Insulin resistance is a pathological condition in which the insulin receptor (IR) is insensitive to insulin and signaling pathway cannot be sufficiently activated. Compared with HFD group, insulin stimulation led to the activation of IR in ginsenoside Rg1-treated group in a dose-dependent manner, as monitored by detection of liver and muscle extracts with an anti-pY1162/3 IR antibody (Figure 2E-H). Taken together, these data suggested that ginsenoside Rg1 improved insulin resistance by increasing sensitivity of IR in HFD mice.

### AMPK pathway was activated in ginsenoside Rg1-treated HFD group

**Figure 3.**
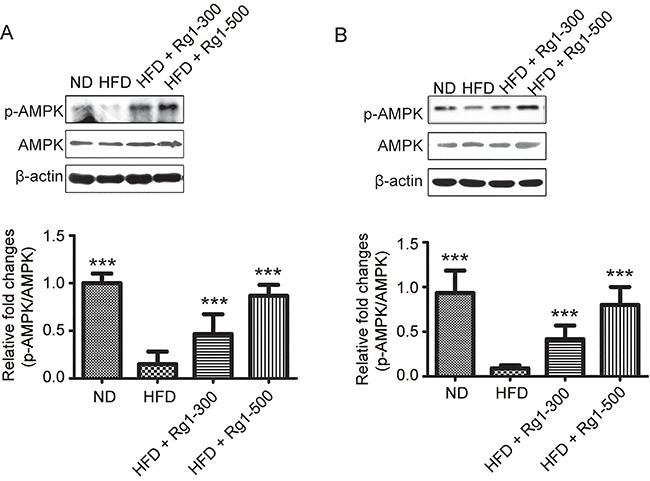
Effect of ginsenoside Rg1 on the AMPK pathway. The high-fat diet (HFD)-fed mice were treated with or without ginsenoside Rg1 at a dose of 300 mg·kg^-1^·day^-1^ (Rg1-300) or 500 mg·kg^-1^·day^-1^ (Rg1-500) for 2 months (n=12 mice/group). Immunoblotting assays detected the basal protein and phosphorylation levels of AMPK in liver (*A*) and muscle (*B*). Results are representative of 2 of 12 mice per group (upper panels). The quantified data are shown in the lower panels. ND: normal diet. Data are reported as means±SD. ***P<0.001, *vs* HFD group (ANOVA).

**Figure 4.**
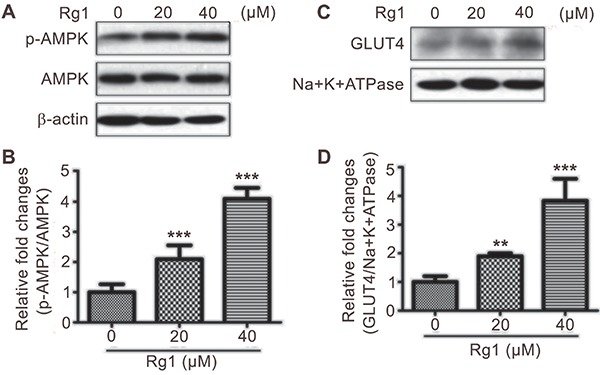
Effect of ginsenoside Rg1 on activation of the AMPK pathway (*A* and *B*) and membrane translocation of GLUT4 (*C* and *D*). C2C12 skeletal muscle cells were treated with ginsenoside Rg1 at concentrations of 0, 20, and 40 μM for 12 h. Data are reported as means±SD of three independent experiments. **P<0.01, ***P<0.001, *vs* control group (ANOVA).

The AMPK pathway is an important regulatory pathway of glucose homeostasis (7). Therefore, we detected activation of the AMPK pathway in mice that were treated or not with ginsenoside Rg1 for two months. The results showed that phosphorylation of AMPK was lower in liver and muscle of HFD mice than that of the ND mice, whereas compensating effects were observed in the ginsenoside Rg1 treatment group (Figure 3). Furthermore, our results showed that ginsenoside Rg1 treatment also activated the AMPK pathway in C2C12 skeletal muscle cells in a dose-dependent manner (Figure 4A and B). Taken together, these results suggested that ginsenoside Rg1 activated the AMPK pathway *in vitro* and *in vivo*.

### Membrane expression of GLUT4 was increased in HFD mice treated with ginsenoside Rg1

GLUT4 translocation to the plasma membrane is an important regulatory factor of glucose uptake (5,20). To explore the molecular mechanism of ginsenoside Rg1-regulated glucose intolerance and insulin resistance, we further detected the membrane expression of GLUT4 in C2C12 skeletal muscle cells treated with ginsenoside Rg1. We observed that the membrane expression of GLUT4 is upregulated by ginsenoside Rg1 treatment in a dose-dependent manner (Figure 4C and D). Thus, these observations suggested that ginsenoside Rg1 improved insulin resistance and glucose intolerance by up-regulating translocation of GLUT4 from intracellular pool to the plasma membrane.

## Discussion

Dietary-induced obesity is the most common type of obesity (18). Obesity is one of the most important risk factors for T2D. Currently, obesity and T2D greatly threaten global population health (21–23). Although various treatments have been used in clinical therapy of obesity including surgical intervention, medication, restriction of food intake, and increasing physical exercise, these therapeutic approaches have some limitation such as serious side effects, poor long-term adherence rates, and inefficiency in some types of obesity (24,25). Thus, new treatments for obesity and its related glucose metabolic disorders are urgently sought. It has been reported that ginseng extracts have inhibitory effects on obesity and diabetes (13). Ginsenoside Rg1 is a bioactive component of ginseng. Here, we investigated the anti-obesity and anti-diabetic effects of ginsenoside Rg1 in an animal model.

In this study, we investigated the effect of ginsenoside Rg1 on dietary-induced obesity. Our results showed that ginsenoside Rg1 impaired body weight gain and fat accumulation in WAT of HFD-fed mice. These findings are in accordance with previous reports that ginseng extracts prevent obesity in HFD mice and in an obese insulin-resistant rat model (9,13). Furthermore, we found that ginsenoside Rg1 activated the AMPK pathway in HFD mice, which was consistent with previous studies (14,26). AMPK plays an important role in maintenance of glucose energy homeostasis in whole-body level and is a key regulator of obesity (27). AMPK activation induces cellular catabolism of lipids, protein and sugars via multiple downstream pathways (28). Specifically, AMPK impedes mTOR complex 1 (mTORC1) activity (29,30). When mTORC1 were specifically knocked out in adipose tissue, mice exhibited the phenotype of resistance to dietary-induced obesity (31). Considering our results and previous observations together, it is suggested that ginsenoside Rg1 inhibits dietary-induced obesity by activation of AMPK/mTORC1 pathway.

Furthermore, we found that ginsenoside Rg1 decreased fasting and postprandial blood glucose level and alleviated insulin resistance in HFD mice. These observations agree with previous reports that ginseng extracts improve insulin resistance in rat and ginsenoside Rg1 increases glucose uptake (9,14,32). Glucose transport is regulated by the PI3K/AKT pathway and the AMPK pathway. When insulin binds to IR, IRS, and the PI3K/AKT pathway is activated, which leads to GLUT4 translocation to the plasma membrane, and then induces glucose uptake (5,20). Our results showed that ginsenoside Rg1 activated IR and the AMPK pathway in skeletal muscle and liver of HFD mice as well as increased GLUT4 translocation to the plasma membrane in C2C12 skeletal muscle cells. However, Lee et al. (14) have reported that ginsenoside Rg1 has no effect on the PI3K/Akt signaling pathway in muscle cells. A possible explanation for the contradictory observations of insulin pathway activation in cellular level and animal level is the effects of ginsenoside Rg1 on insulin resistance and glucose uptake, which could be an indirect secondary effect to its anti-obesity function. Meanwhile, AMPK directly induced GLUT4 translocation to the plasma membrane independent of the PI3K/Akt pathway (33), which is similar to the results of our study. Thus, our data also suggested that ginsenoside Rg1 increased glucose uptake as well as improved insulin resistance and glucose intolerance by activating AMPK signaling.

In conclusion, we have demonstrated that ginsenoside Rg1 protected mice from dietary-induced obesity and its related glucose metabolic disorders via activating the AMPK pathway. Therefore, ginsenoside Rg1 might be a natural bioactive compound that has anti-obesity and anti-diabetic properties in an animal model.
